# Investigating the Performances of Wide-Field Raman Microscopy with Stochastic Optical Reconstruction Post-Processing

**DOI:** 10.1177/00037028211056975

**Published:** 2022-02-05

**Authors:** Leila Mazaheri, Joachim Jelken, María O. Avilés, Sydney Legge, François Lagugné-Labarthet

**Affiliations:** Department of Chemistry, The Centre for Advanced Materials and Biomaterials Research (CAMBR), 195984 The University of Western Ontario (Western University), London, ON, Canada

**Keywords:** Wide-field Raman imaging, hyperspectral Raman imaging, stochastic optical reconstruction microscopy, localizing microscopy

## Abstract

Super-resolution fluorescence microscopy based on localization algorithms has tremendously impacted the field of imaging by improving the spatial resolution of optical measurements with specific blinking fluorophores and concomitant reduction of acquisition time. In vibrational spectroscopy and imaging, various methods have been developed to surpass the diffraction limit including near-field scattering methods, such as in tip-enhanced Raman and infrared spectroscopies. Although these scanning-probe techniques can provide exquisite spatial resolution, they often require long acquisition times and tedious fabrication of nano-scale scanning probes. Herein, stochastic optical reconstruction microscopy (STORM) protocol is applied on Raman measurements acquired using a wide-field home-built microscopy setup. We explore how the fluctuations of the Raman signal acquired over a series of time-lapse images at specific spectral ranges can be exploited with STORM processing, possibly revealing details with improved spatial resolution, under lower irradiance and with faster acquisition speed that cannot be achieved in point scanning mode over the same field of view. Samples studied here include patterned silicon, polystyrene microspheres on a silicon wafer, and graphene on a silicon/silicon dioxide substrate. The outcome presents an effective way to collect Raman images at selected spectral ranges with spatial resolutions of ∼200 nm over a large field of view under 532 nm excitation together with an acquisition speed improved by two orders of magnitude and under a significantly reduced irradiance compared to confocal laser scanning acquisition.

## Graphical abstract

**Graphical Abstract:**
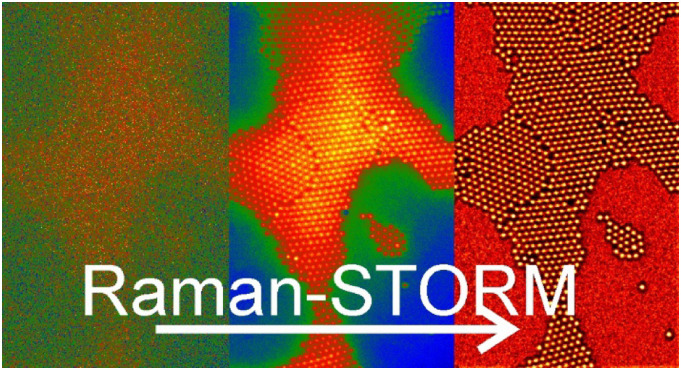

## Introduction

The lateral spatial resolution δ in optical microscopy is inherently limited by the Rayleigh criterion to $\text{δ}=\frac{0.61\text{λ}}{\text{NA}}$ , where λ is the wavelength of observation and NA is the numerical aperture of the microscope objective.^1^ This spatial resolution criterion is based on the distinction of two objects separated by δ and corresponds to a half-Airy unit. On the other hand, the Abbe criterion is given by $\text{δ}=\frac{0.5\text{λ}}{\text{NA}}$ where δ is the smallest resolvable feature. In ideal optical conditions, these physical principles and definitions of spatial resolution limit the spatial resolution of optical microscopy imaging to roughly 200–300 nm when using a visible source. This criterion can be further improved by a $\sqrt{2}$ factor when used in conjunction with a confocal pinhole. To go beyond this physical limit and achieve sub-wavelength resolution, different approaches have been developed, such as near-field scanning optical microscopy (NSOM) and single-molecule localized microscopy (SMLM).

Near-field scanning optical microscopy, used either in collection-transmission or scattering modes, exploits the properties of an evanescent wave confined at the extremity of a scanning probe tip.^2,3^ NSOM has numerous challenges such as limited tip lifetime, poor reproducibility of experiments, long scanning times, and limitation to surface measurements. On the other hand, the development of noninvasive far-field microscopy adopted for super-resolution has significant advantages in microscopy studies and has enabled the possibility of imaging complex biological systems over a wide field of view and with fast acquisition rates.^4^ In this context, SMLM exceeds sub-diffraction spatial resolution by localization reconstruction approaches that use specific blinking chromophores.^5^ Stochastic optical reconstruction microscopy (STORM) is one of these SMLM techniques and highlights a spatial resolution of a few nanometers down to the 3D localization of dense clusters composed of a few emitting molecules, which is of key interest for the ultra-precise imaging of biological systems where cellular labeling is possible.^5,6^

Stochastic optical reconstruction fluorescence microscopy is based on the stochastic photo-switching^7^ and photoactivation^8^ of fluorescent molecules between dark and fluorescent states.^9^ A small subset of fluorophores is consecutively activated, and a series of diffraction-limited images is processed with centroid localization algorithms. A high-resolution image is then reconstructed with mathematical fitting of the temporal fluctuation of the fluorophores via the whole series of time-lapse images. A common evaluation method of localization microscopy is with a Gaussian model of the point spread function (PSF) and a Poissonian maximum likelihood estimator (MLE) fitting of the Gaussian model.^10^

Despite its diffraction-limited spatial resolution, Raman confocal microscopy plays a key role in studying materials and biomaterials with micro- and nano-scale features. In the field of materials, it provides critical information such as the presence of impurities and structural defects,^11^ doping^12^ behavior when subjected to strain–stress force,^13,14^ and the number of layers in the case of 2D materials.^15^ Raman imaging is generally hindered by a weak Raman scattering cross-section, resulting in low signal intensity and thus requiring long acquisition times.^16^ The Raman scattering cross-section is notably large for a wide variety of semiconductor materials used in the electronic industry and for emerging materials such as carbon-containing materials (i.e., carbon nanotubes and graphene) and 2D transition metal dichalcogenides. A variety of approaches are used to amplify Raman signal, including surface-enhanced Raman spectroscopy (SERS),^17^ and tip-enhanced Raman spectroscopy (TERS), which also yields an increase in spatial resolution.^18,19^ However, in TERS, image acquisition collected through a scanning probe is hindered by the long acquisition time of a hyperspectral map.^20^

More recently, Ayas et al. and Olson et al. have applied STORM post-processing on Raman images with the goal of enhancing the spatial resolution using SERS substrates.^21–23^ In particular, Olson et al. use the field fluctuations from a plasmonic substrate to locally enhance the contrast of the object yielding a spatial resolution of ∼10 nm.^22,23^ However, the authors were integrating over all Raman modes and background and were not imaging a specific vibrational mode but rather a background fluctuation, losing the molecular specificity of Raman spectroscopy.^22^

Building on Olson’s work, we have investigated the possibility of wide-field Raman imaging with stochastic optical reconstruction post-processing of time-lapse Raman images, suggesting that the Raman imaging system can evolve towards high resolution measurements with a higher speed over larger field of view and under a lower irradiance. Such an approach could be used for the quality control of electronic chip production, revealing the presence of defects in semiconductors or photonic circuits and more generally for materials with large scattering cross sections.^24^

In this work, we have developed a wide-field Raman imaging microscope that integrates a set of liquid crystal tunable filter and edge filters to select relevant spectral domains corresponding to selected Raman modes. The spatial resolution was further evaluated with STORM treatment over a series of dynamically acquired wide-field Raman images. Samples explored in this work include patterned Si and Si/SiO_2_, polystyrene microspheres deposited on an Si substrate, and exfoliated graphene on an Si/SiO_2_ substrate. Other nanostructures of Au and Si were imaged to gage the resolution and the speed of the acquisition.

## Experimental

**Figure 1.**
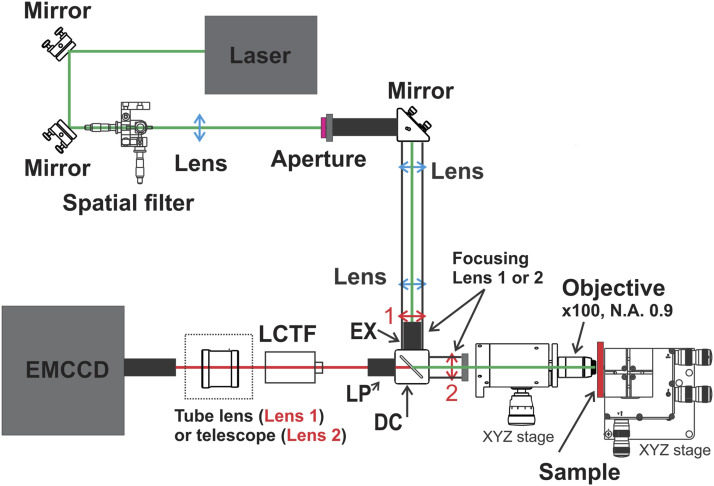
Schematic of the wide-field hyperspectral Raman imaging system. The excitation beam is presented in green and Raman signal in red. OD: optical density filter, EX: excitation filter LP: long-pass filter, DC: dichroic mirror, LCTF: liquid crystal tunable filter, EMCCD: electron multiplication charge-coupled devices. Lens 1 is used in conjunction with a tube lens, whereas Lens 2 is used in conjunction with a telescope.

The microscope developed here is entirely built from optical and optomechanical elements in a horizontal geometry as pictured in Fig. 1 and does not use a commercial microscope body. A continuous wave 532 nm laser (Opus, Laser Quantum, with power up to 4 W) is first cleaned using a spatial filter (10× objective and 20 μm pinhole) and collimated with the help of a plano–convex lens (focal length 60 mm) resulting in a parallel beam of about 1 cm diameter. An excitation filter is used to remove any spurious lines from the laser (Chroma, RET532/4×). The beam passes through a dichroic beam splitter (Chroma, RT532rdc), which reflects light at 532 nm and transmits longer wavelengths. A lens of focal length 25 cm (Lens 1) or 17.5 cm (Lens 2) focuses the beam on the back focal plane of the 100× Olympus objective (M Plan Semi Apochromat (MPLFLN); NA = 0.9). The working distance is 1 mm with the back focal plane located 8 mm away from the back flange of the microscope objective. The full field of view of the objective is a circular area with a diameter of 265 µm. The typical field of view projected onto the detector is (110 × 110) μm^2^. The sample and objective are placed on *x*,*y*,*z* micrometer translation stages (Thorlabs) that enables precise objective and sample positioning. The scattered light is collected and collimated with the same objective in the backscattering geometry. Two long-pass filters (Chroma, RET537lp) are used to suppress the Rayleigh scattering of the laser. The spectral range of Raman scattering is selected with a liquid crystal tunable filter (LCTF, Thorlabs, KURIOS-VB1). The minimum bandwidth of the LCTF is limited to 10 nm (i.e., 300 cm^−1^) for the highest spectral resolution. Finally, when used in conjunction with Lens 1, a tube lens (TTL180-A) is used to direct the Raman image into the electron multiplication charge-coupled devices (EMCCD; Ixon ultra 897 Andor, 512 × 512 pixels). When used with Lens 2, a telescope was used instead of the tube lens. Raman images are acquired at 56 fps. The CCD was thermoelectrically cooled to –50 °C and images are back-illuminated with quantum efficiency higher than 90%. To achieve optimal signal-to-noise ratio and faster performance, the EMCCD is operated at 1 MHz readout speed in frame transfer mode, simultaneously acquiring images while reading out previous images from the storage area, thereby wasting no time between different frames. The images are recorded using the Andor Solis software system.

For all images presented in this paper, the maximum laser power is set to 1.5 W, yielding an irradiance of 2.7 × 10^3^ W/cm^2^ at the sample plane. A single image is typically acquired in 0.28 s over the whole field of view. In order to compare the performance of our measurements, reference Raman images and spectra over the same samples were collected on a Raman confocal microscope with a liquid N_2_-cooled charge-coupled device (CCD) and confocal pinhole of 200 µm (LabRAM HR, Horiba Scientific) and a Raman confocal microscope with a Peltier-cooled EMCCD and confocal pinhole of 100 µm (XploRA Plus, Horiba Scientific). In these measurements, a 532 nm laser of 20–30 mW was focused on the sample with a 100× Olympus objective (MPLFLN, NA = 0.9), yielding an irradiance of 2 × 10^6^–3 × 10^6^ W/cm^2^ at the focal point (∼1 μm^2^ spot) and a spectral resolution of 3 cm^−1^. We note here that the irradiance for the point scanning experiments is three orders of magnitude higher when compared to the wide-field imaging. The sample was raster scanned with an (*x*,*y*) motorized stage.

## Results and Discussion

In conventional Raman mapping, the excitation wavelength is focused on the sample with a typical spot size of 1 µm when using high NA objectives. The sample is then raster scanned, yielding a hyperspectral cube in which a spectrum is acquired on each set of (*x*,*y*) coordinates to capture an image for a large area. An alternative for imaging a large area is to homogeneously illuminate the full field of view (FOV) and image the sample over areas that are typically hundreds of µm^2^ in size. However, the application of wide-field imaging for vibrational spectroscopy is limited by the performance of the filtering system used to select a spectral domain. Recent work on Raman wide-field imaging using a Bragg tunable filter (BTF) has shown great results due to the narrow bandwidth (8 cm^−1^) and tunability over a large spectral domain.^25^ The angular selectivity of the BTF leads to a wavelength gradient over the image produced on a CCD which is followed by numerical reconstruction to extract the full FOV at a given Raman wavenumber. Another approach relies on selecting the spectral range of the scattered light over the full FOV, using filtering schemes like liquid crystal tunable filters.^26,27^ The potential of imaging the Raman FOV facilitates a fast and easy method for material characterization that also allows for far-field spatial resolution. However, the low throughput of available filters and the broad bandwidth limit the use of these systems. One additional issue to consider in wide-field illumination arises from the fluorescence of the objective lenses since the laser beam is focused at the back focal plane of the objective. This fluorescence can generally be treated as a background and does not limit the sensitivity of the system when Raman modes are intense and well-contrasted.

Coupling label-free Raman imaging with stochastic microscopy is a potential alternative for chemical fingerprint imaging.^21,28^ The dynamic nature of the hotspot generation produces time-dependent SERS signal^29–33^ that can be further exploited for STORM processing. However, using SERS for wide-field imaging requires a uniform and dense distribution of hotspots which is difficult to achieve experimentally.

Herein, instead of using a SERS substrate, wide-field Raman images over conducting, semi-conducting, and dielectric surfaces were acquired over time-lapse sequences of hundreds to thousands of images. The temporal fluctuation of the Raman scattering was then localized with open source RapidSTORM software. This spontaneous intensity fluctuation is not controllable in similar fashion to shifting hotspots or photo-switching fluorophores. The origin of the Raman fluctuation can be related to molecular adsorption–desorption, surface diffusion, or molecular reorientation at the surface in ambient conditions.^34^ In the case of conductive or semi-conductive surface, the fluctuations can also arise from the generation and rearrangements of local hot-spots within picocavities.^33,35^ Signal fluctuations may also come from thermal effect and laser intensity fluctuations. In wide-field illumination, the probability of inducing vibrational transition and Raman scattering for each point of the sample can differ depending on the absorption of light. Therefore, analyzing the fluctuations for each pixel of the 2D detector recorded over a series of wide-field images can provide a snapshot of the areas with larger Raman intensity fluctuation.

### Raman-STORM of Silicon Structures

**Figure 2.**
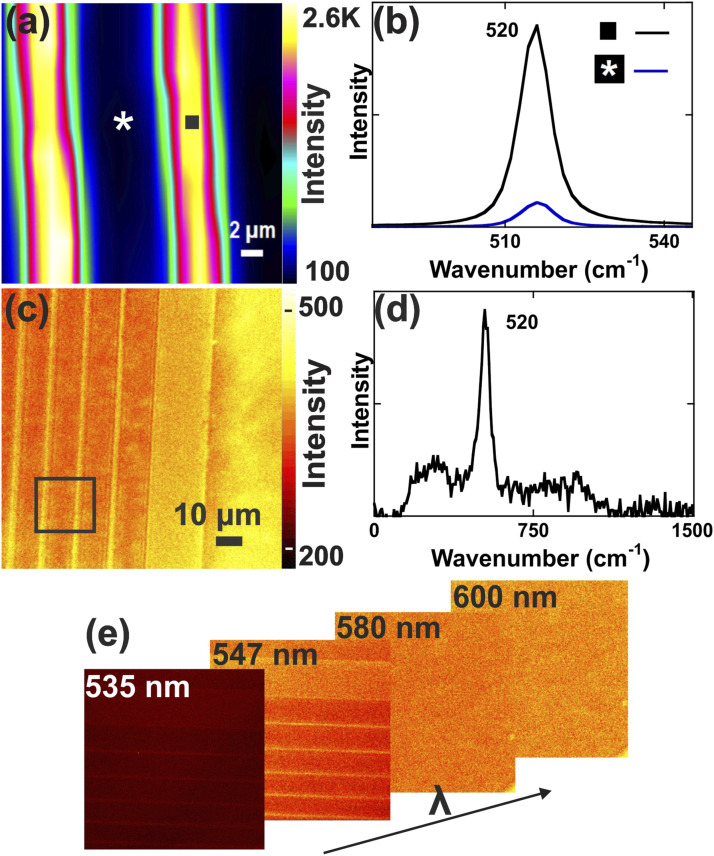
(a) Confocal Raman mapping 25 × 15 µm^2^ of a patterned Si obtained in point scanning mode and (b) corresponding spectra, where the blue spectrum corresponds to dark area highlighted with an asterisk in (a) and the black spectrum corresponds to the bright area highlighted with a black square in (a). (c) 110 × 110 µm^2^ wide-field Raman image collected in 0.28 s, where the rectangle highlights the area where confocal scanning was done in (a). (d) Si spectrum acquired by the wide-field system. (e) Collected images of the patterned Si sample acquired at central wavelengths of 535, 547, 580, and 600 nm, corresponding to spectral regions of –70–279, 347–682, 1406–1703, and 1991–2268 cm^–1^, respectively.

Figure 2a is Raman map of a patterned Si structure collected with a confocal Raman microscope that represents the variation in intensity of the Si phonon mode at 520.7 cm^–1^. This point-mapping result is presented for an area of 25 × 15 μm^2^, representing 1000 individual Raman spectra collected in 30 min. The intensity of the Si Raman peak for dark and brighter lines differs by factor of 20 (Fig. 2b). On the same sample, a single wide-field Raman image centered at 520 cm^–1^ and over a 300 cm^–1^ spectral range (narrow settings) is shown in Fig. 2c and was captured in 0.28 s. The FOV is much larger in the wide-field images compared to the confocal images, as a 110 × 110 μm^2^ image was obtained by the former and a 25 × 15 μm^2^ image was obtained by the latter. The background was subtracted using a polynomial baseline function. The Si spectrum was also collected on the wide-field microscope with the addition of a spectrometer after the long-pass filters (Fig. 2d). The scattered light from the wide-field image was focused into a fiber-coupled spectrometer (Ocean Optics Inc., USB 4000), revealing the Raman spectrum of Si and the absence of Rayleigh scattering. For the wide-field imaging, the 1.5 W excitation laser beam was focused on the back focal plane of the objective and resulted in a background due to the fluorescence of the objective lens. To correct this background for Raman imaging, the response of the EMCCD was measured without the sample and under similar acquisition time and laser power. The background correction was then stored in the EMCCD acquisition software.

The tunable bandpass filter allows for the continuous tuning of the central wavelength from 420 to 730 nm with a narrow bandwidth of 10 nm (300 cm^−1^). To evaluate the potential of hyperspectral imaging based on wavelength scanning for full FOV, the patterned Si sample was imaged at different wavelengths (Fig. 2e). At the central wavelength of 535 nm, a low contrast image was captured, corresponding to the background. The image with the LCTF filter centered at 547 nm yielded the highest contrast since it corresponds to the 520.7 cm^−1^ mode of Si. Other spectral domains collected at 580 nm and 600 nm do not show any contrast as expected.

Raman images of the patterned Si with RapidSTORM post-processing are presented in Fig. S1 (Supplemental Material). The minimum spot distance (distance between the maximum of two Gaussian PSF with a constant FWHM defined by the optical system and set to 500 nm in the software, which corresponds to approximately four pixels of the EMCCD) and intensity threshold values (minimum intensity until which fluctuations are getting considered) are two important parameters utilized in the RapidSTORM software in order to fit the dynamic series and to localize the highest Si contrast while reducing the background contribution.^36,37^ The spatial distribution of the localized spots yields a crisper contrast for the Si lines with a minimum spot distance of one pixel, which corresponds to a dimension of ∼130 nm dependent on the telescope used in the setup, and threshold intensity of five (Fig. S1a).

### Raman-STORM of Polystyrene Microspheres

**Figure 3.**
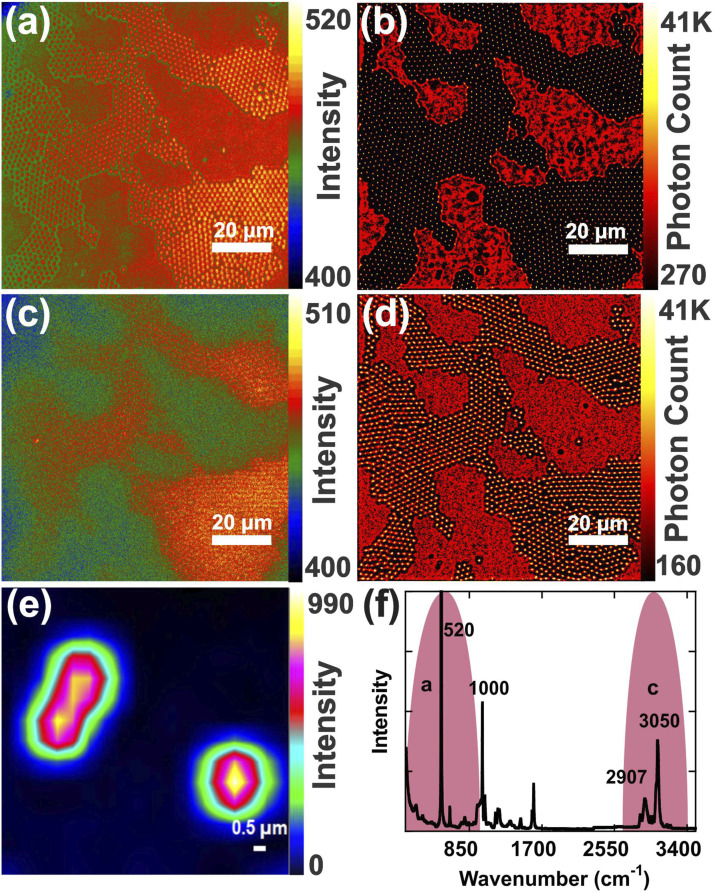
Wide-field Raman imaging of 2 µm PS spheres on an Si substrate alongside RapidSTORM images reconstructed from a series of 100 images (28 s total acquisition time). (a) Single wide-field image integrated over the 200–1004 cm^–1^ spectral range and (b) corresponding Raman-STORM image. (c) Single wide-field image integrated over the 2668–3413 cm^–1^ spectral range and (d) corresponding Raman-STORM image. (e) 23 × 19 point Raman map of a single PS sphere and two clustered PS spheres acquired by the confocal system with a 0.5 µm step along the *x*-axis and 3 s acquisition per point (20 min total acquisition time). The map is integrated over the 505–535 cm^–1^ spectral range. (f) Raman spectrum of PS highlighting the spectral ranges used in (a–d).

Raman-STORM was used to image a monolayer of polystyrene (PS) spheres of diameter 2 µm organized onto a Si substrate. Figure 3 presents the images captured in the wide setting of the LCTF with 30 nm bandwidth. Figure 3a covers the 200–1004 cm^−1^ spectral range, where the Si mode at 520 cm^−1^ has the highest intensity. This spectral range overlaps with Raman peaks of PS at 620, 795, and 1000 cm^−1^. However, due to the Gaussian transmission profile of the LCTF, the signal of the second order Si phonon at 1000 cm^−1^ as well as ring mode of PS will have a smaller scattering cross section. The bands at 620 cm^–1^ and 795 cm^–1^ are also very weak compared to the Si signal at 520 cm^–1^. The centers of the PS spheres appear brighter in this spectral region since the spheres act like micro-lenses and focus the beam on the Si substrate. The Raman signal enhancement by the lens effect of the PS spheres, due to its high refractive index of 1.59,^38^ was illustrated for confocal microscopy.^39^ Raman signal intensity correlates to the incident intensity and the square of the numerical aperture of the objective.^40^ The lens effect induces another focusing system, thus increasing the numerical aperture of the system and the resulting intensity.^39^ The combination of the PS lens effect and the wide-field excitation provides a potential benefit in Raman spectroscopy studies due to the resulting higher intensity Raman signal and larger FOV.

In the reconstructed STORM image, the bare Si area has a positive contrast while the centers of the PS spheres appear as bright dots (Fig. 3b). The location of the PS spheres can be determined with higher precision from the reconstructed STORM image compared to the wide-field image (Fig. 3a). Conversely, in the Raman image at 2668–3413 cm^–1^ corresponding to CH stretch bands in PS at 3050 cm^–1^ at the central wavelength of 635 nm, the area covered with PS spheres has a positive contrast compared to the Si substrate (Fig. 3c). The PS spheres appear more defined with more precise locations in the STORM image (Fig. 3d) compared to the wide-field Raman image (Fig. 3c). For comparison, a Raman confocal map of one and two PS spheres integrated over 505–535 cm^–1^ was acquired in 20 min (Fig. 3e). Comparing the Si signal on bare Si with the area covered by PS spheres in point scanning confirms the lens effect, since the Si signal is enhanced by a factor of 1.4. The Raman spectrum of the PS spheres on the Si substrate is presented in Fig. 3f, in which the selected spectral ranges for wide-field imaging are specified.

The spectral resolution of the wide-field imaging system is restricted by the bandwidth of the tunable liquid crystal cell. Figure S2 shows the comparison of the wide and narrow settings of the LCTF for PS sphere imaging at a central wavelength of 562 nm. Once processed with STORM, the poorly contrasted Raman wide-field images provided sharper contrast. The treatment of 100 dynamic images yielded significantly crisper images when compared to a single image for both wide and narrow spectral settings (Figs. S2a–d).

**Figure 4.**
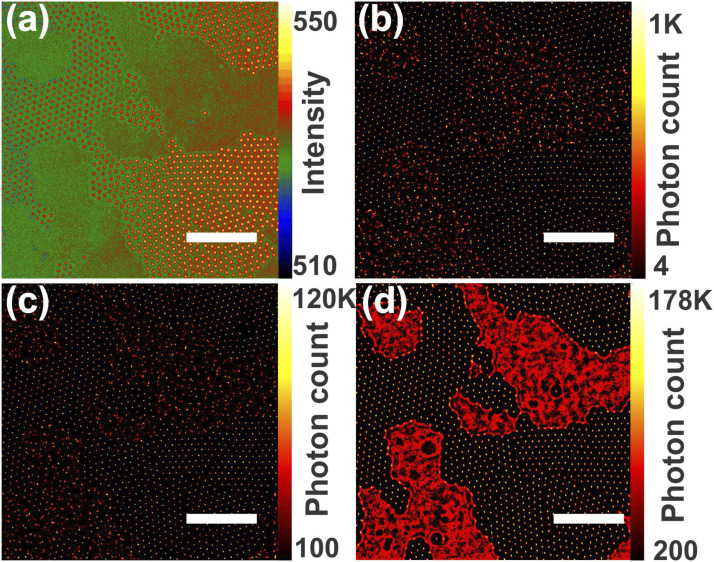
(a) Wide-field Raman image of 2 µm PS spheres on an Si substrate at 200–1004 cm^–1^, and RapidSTORM-treated images of (b) a single image, (c) a series of 100 identical images, and (d) a series of 100 kinetic images (scale bars = 20 μm).

For comparison, the STORM process was applied to a single image, a series of 100 identical images, and a dynamic series of 100 images (Fig. 4). For reference, a single wide-field Raman image is provided (Fig. 4a). Post-processing of a single image is identical to stationary scattering (Fig. 4b) and only highlights the very intense pixels. To illustrate the importance of the temporal oscillation, a series of 100 identical images was created by cloning a given image and further treated with RapidSTORM (Fig. 4c). In this stationary stack of images, higher intensity spots get localized, yielding similar results to Fig. 4b. On the other hand, a kinetic series of 100 images provides temporal oscillations of Raman signal which greatly improves the quality of the final image, since the centers of the PS spheres and the distribution of the Raman fluctuations on the bare Si surface are clearly visible (Fig. 4d).

To further evaluate the signal-to-noise enhancement from the STORM process, the Raman-STORM images are compared to an averaged ensemble of collected images (Fig. S3). A kinetic series of 500 images treated with RapidSTORM (Fig. S3a) and an image averaged from the accumulation of 500 images (Fig. S3b) are compared. For both approaches, the whole series of images are acquired in 140 s. STORM post-processing remarkably enhances the contrast and the uniformity of the Raman image.

**Figure 5.**
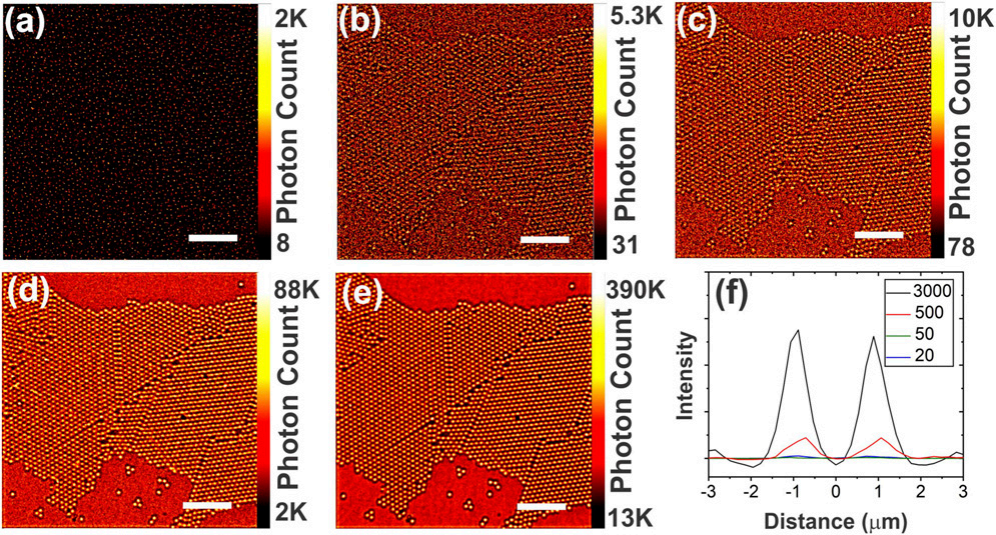
(a) Single image collected with LCTF at 635 nm, corresponding to a spectral range of 2668–3413 cm^–1^, and RapidSTORM-treated images of a kinetic series of (b) 20, (c) 50, (d) 500, and (e) 3000 images (scale bars = 20 µm). (f) Optical cross-section of two side-by-side PS spheres.

The STORM images are presented in Figs. 5a–e for a different number of acquired images of 2 µm PS spheres, with the number of images varying from 1 to 3000. Larger image series (Figs. 5d and 5e) yield increased contrast compared to smaller image series (Figs. 5b and 5c). With 500 images, the details of the STORM image are fully formed (Fig. 5d). Increasing the number of images to 3000 further increases the sharpness and contrast of the STORM image (Fig. 5e). The corresponding optical cross-section for two side-by-side PS spheres highlights the improved signal-to-noise ratio achieved with a larger number of collected images (Fig. 5f).

### Raman–STORM of Graphene Layers

**Figure 6.**
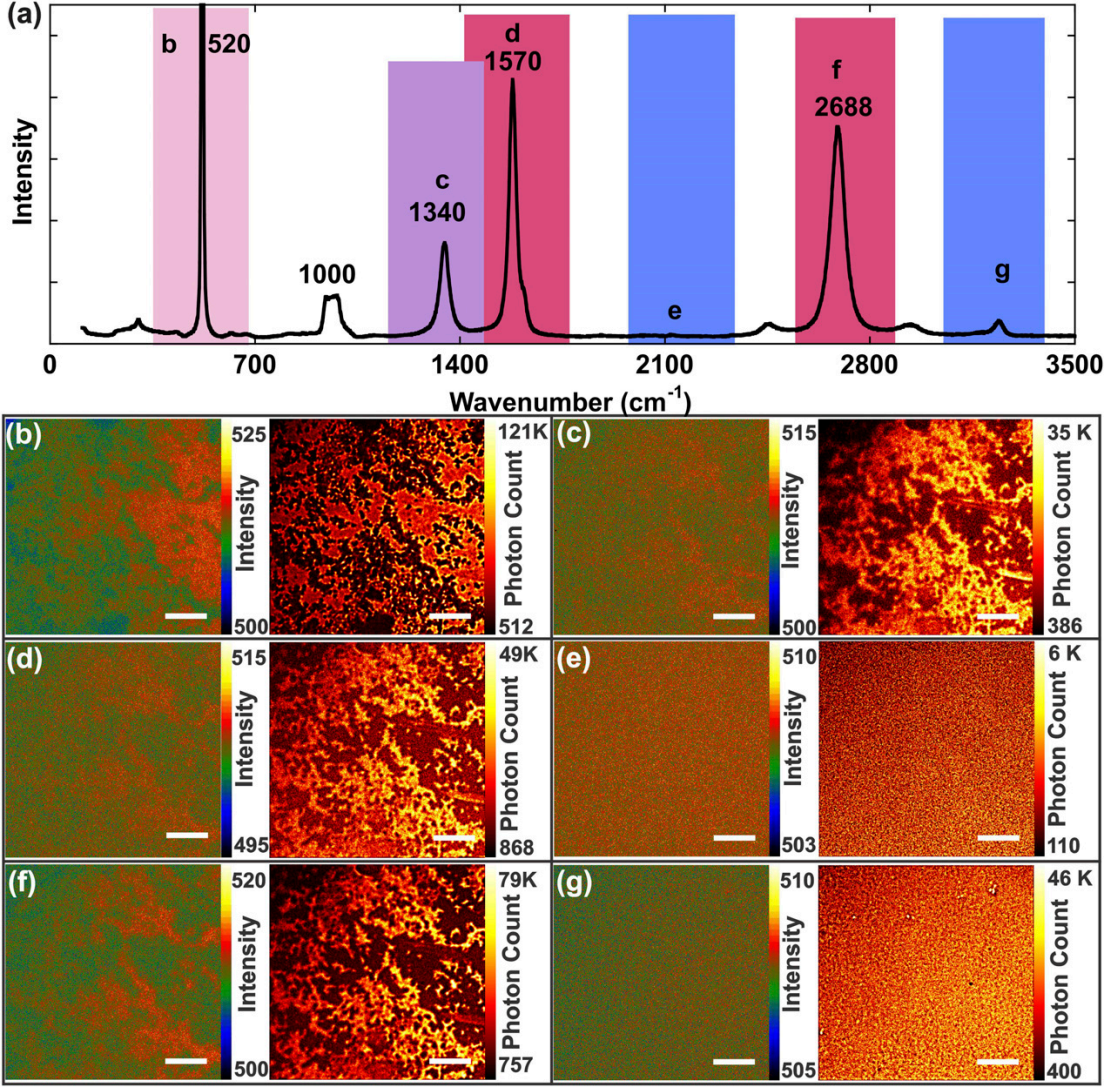
(a) Raman spectra of exfoliated graphene deposited over an Si/SiO_2_ substrate. 110 × 110 μm^2^ wide-field Raman images of a solution of graphene/ethanol drop-casted on an Si/SiO_2_ substrate at a central wavelength of (b) 547 nm (347–681 cm^–1^), (c) 573 nm (1192–1496 cm^–1^), (d) 580 nm (1406–1703 cm^–1^), (e) 600 nm (1963–2413 cm^–1^), (f) 620 nm (2537–2797 cm^–1^), and (g) 640 nm (3049–3293 cm^–1^), alongside their RapidSTORM-treated images from 500 images (scale bars = 20 μm).

Graphene is an ideal sample for Raman measurements due to its large intrinsic Raman scattering cross section and it is often used as a benchmark material for wide-field Raman imaging.^25,41^ The Raman spectrum of a graphene sample deposited on an Si substrate is shown in Fig. 6a. Briefly, the D band (1340 cm^–1^) corresponds to disorder in the sp^2^ lattice of graphene. The G band (1580 cm^–1^) is the main Raman band assigned to the in-plane sp^2^ C–C stretching mode. The 2D band is the second order Raman process representing the in-plane breathing mode of the carbon rings.

The wide-field set of Raman images of a liquid exfoliated solution of graphene/ethanol drop-casted on an Si/SiO_2_ substrate were acquired and a series of 500 images were post-processed with RapidSTORM in distinct spectral domains (Figs. 6b–6g). Raman maps were acquired at a central wavelength of 547 nm, corresponding to a spectral region of 347–681 cm^–1^ and thus integrating the Si Raman phonon mode (Fig. 6b). Other integrated regions centered at 573 nm or 1192–1496 cm^–1^ (Fig. 6c), 581 nm or 1406–1703 cm^–1^ (Fig. 6d), and 620 nm or 2537–2797 cm^–1^ (Fig. 6f) correspond to the D, G, and 2D Raman bands of graphene, respectively. An opposite contrast is seen when the graphene modes are integrated (Figs. 6c, 6d, and 6f) compared to when the Si mode is integrated (Fig. 6b). Wide-field images collected outside the regions with intense Raman modes at 600 nm (Fig. 6e) and 640 nm (Fig. 6g) do not show any vibrational contrast, as expected.

### Estimation of the Spatial Resolution Improvement from STORM Processing

**Figure 7.**
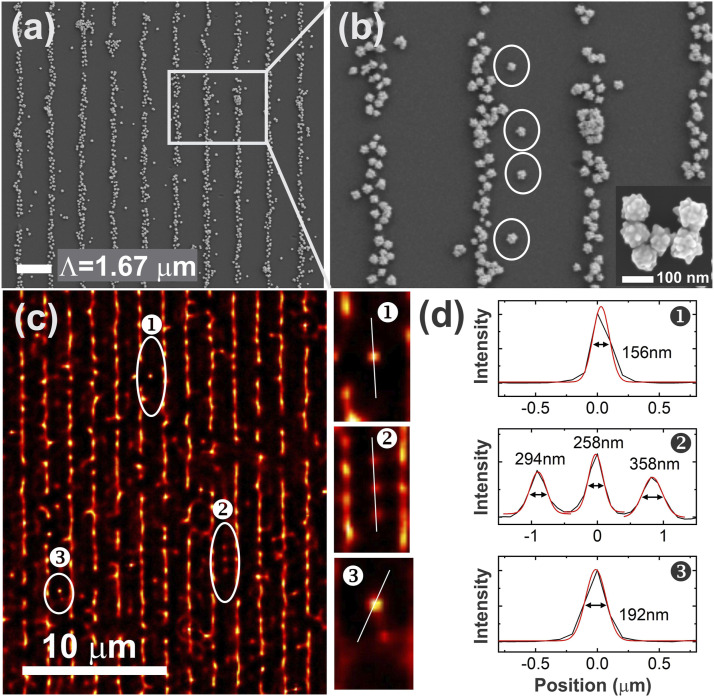
(a) SEM image of Au nanoraspberries organized along parallel lines after imprinting onto a glass coverslip. (b) Higher-magnification SEM image of the rectangular area in (a) highlighting isolated nanoraspberries with sizes of 80–100 nm. (c) Image treatment by RapidSTORM from a kinetic series of 1000 images. (d) Cross section of isolated particles highlighted in (c) with FWHM values.

The spatial resolution of a microscope image relates to both the optical resolution of the microscope and the digital spatial density of the images. The optical resolution relates to the diffraction limit, numerical aperture, and the quality of the optical setup, whereas the spatial density is related to the number of pixels of the detector and the distance between two pixels. Considering all the influencing features, the spatial resolution may be greater than λ/2. The spatial resolution of the instrument for imaging and localizing single nanoparticles was evaluated on a sample composed of 80–100 nm diameter Au nanoraspberries patterned along parallel lines with a pitch of 1.67 µm (Figs. 7a and 7b). The samples were prepared on a glass slide by soft lithography using a polydimethylsiloxane stamp replicated from a 600 grooves/mm grating.

STORM treatment enables the localization of isolated individual 100 nm particles as shown by the isotropic scattering of these single particles localized between two grooves (Fig. 7c). Similar isolated particles can be seen on the scanning electron microscopy (SEM) images (Figs. 7a and b). Despite the little contrast shown in a single wide-field image (Fig. S4), the STORM treatment applied over a series of 1000 images (total acquisition time of 280 s) provided sufficient optical contrast to locate single nanoraspberries highlighted with circles on the SEM image (Fig. 7b) and on the STORM treated image (Fig. 7c). The selected particles show homogenous circular localization functions (Fig. 7c), confirming that they are isolated single particles as seen in the SEM images (Fig. 7b).

The full width half-maximum (FWHM) values of the isolated nanoraspberries range from 156 to 358 nm (Fig. 7d). Although the optical resolution of our setup does not allow us to truly image a single 100 nm particle, the estimated resolution from this experiment is slightly better than the Abbe criterion.^42^ For an excitation at 532 nm, one shall expect a diffraction-limited resolution of 295 nm.

**Figure 8.**
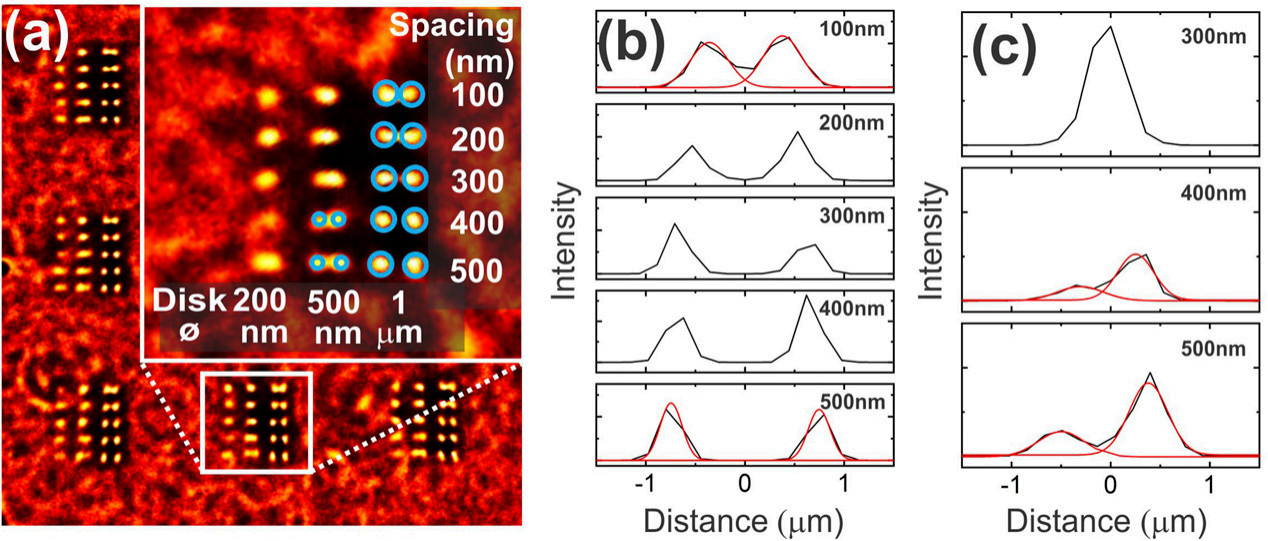
(a) RapidSTORM-treated images of a resolution pattern of Si disks inscribed on an SiO_2_ substrate, consisting of 1, 0.5, and 0.2 µm disks separated by 500, 400, 300, 200, and 100 nm edge-to-edge gap distance (inset). 1000 images were acquired (total time of acquisition 280 s). (b) Cross-sections along two 1 μm Si disks of 100–500 nm spacing and fitting using Gaussian curves for the 500 nm and the 100 nm spaced disks. (c) Cross-sections along two 500 nm Si disks of 300–500 nm spacing and fitting using Gaussian function for the 500 nm and 400 nm spaced disks.

Finally, to further estimate the spatial resolution obtained with our setup, a resolution target consisting of Si disks over a glass substrate were prepared by electron-beam lithography. The diameters of the Si disks were set to 1, 0.5, and 0.2 µm, and their separation (edge-to-edge gap distance) was varied at 500, 400, 300, 200, and 100 nm. 1000 images of the 30 nm-thick Si disk on glass samples were processed with RapidSTORM (Fig. 8a). With STORM processing in narrow settings centered over the Si band at 520 cm^–1^, the 1 μm Si disks could be resolved with a separation distance down to 100 nm (Fig. 8b). For the 500 nm disks, the processing enabled visualization of the disks until a 400 nm separation (Fig. 8c). Interestingly, the localization for even the 1 μm disks appears more confined on the resulting STORM images (Figs. 8b and 8c). From the cross-section analysis, the localization function appears to be similar for both the 1 μm and 500 nm disks (FWHM ∼ 250–300 nm). In both cases, the center-to-center distance correlates well with the physical object but maximum fluctuations appear confined at the centers of the disks.

The resolution experiment was compared with confocal experiments conducted on the same sample (Fig. S5). The confocal experiments were conducted over a 12 × 12 µm^2^ surface using point scanning. The total duration of the experiment was 12 min with an acquisition time of 0.5 s per spectrum. The map in Fig. S5 was integrated over the 494–532 cm^–1^ spectral range. The spatial resolution can be seen along the cross-section of the disk pairs and appears very similar to the STORM-treated images. However, the STORM images were acquired much more rapidly (280 s total for 1000 images) and over a much larger field of view of 110 × 110 µm^2^. A confocal map with the point scanning parameters outlined above over a 110 × 110 µm^2^ surface would take over 18 h, a duration impossible to sustain without any mechanical drift of the sample stage or microscope objective. Such gain of over two orders of magnitude in speed acquisition together with a decrease by three orders of magnitude of the irradiance, as compared to confocal measurements, are therefore of clear interest for specific applications where these two parameters are critical.

## Conclusion

Herein, a wide-field Raman imaging system based on wavelength scanning with liquid crystal tunable filter (LCTF) and single photon sensitive EMCCD with a fast data acquisition rate was described. Coupling stochastic microscopy with wide-field Raman imaging enabled rapid data acquisition and provided crisper contrast compared to confocal microscopy measurements. Time series of wide-field Raman images were greatly improved using STORM post-processing based on several micro- and nano-sized reference samples. Treatment with RapidSTORM was applied to time series Raman images, which showed that temporal fluctuation of Raman scattering can be exploited to provide crisper images over a large field of view with reduced acquisition time and under lower laser power, despite lower spectral resolution due to the limited bandwidth of the LCTF. Importantly, the fluctuations exploited in this process and the results of the STORM treatment applied to the wide-field Raman images also depend on the material studied. While faster acquisition can be conducted by STORM compared to a point scanning experiment over the same area, the spatial resolution is about the same order of magnitude compared to confocal microscopy. We anticipate that an optimized microscope setup with achromatic optics, larger magnification of the image over the detector, and tunable spectral filter with narrower bandwidth would further improve the performance of this approach.

## Supplemental Material

sj-pdf-1-asp-10.1177_00037028211056975 – Supplemental Material for Investigating the Performances of Wide-Field Raman Microscopy with Stochastic Optical Reconstruction Post-Processing Supplemental Material, sj-pdf-1-asp-10.1177_00037028211056975 for Investigating the Performances of Wide-Field Raman Microscopy with Stochastic Optical Reconstruction Post-Processing by Leila Mazaheri, Joachim Jelken, María O. Avilés, Sydney Legge, and François Lagugné-Labarthet in Applied Spectroscopy
