# Supplementary material for: Investigating the Performances of Wide-Field Raman Microscopy with Stochastic Optical Reconstruction Post-Processing
Source: Appl Spectrosc. 2022 Feb 5;76(3):340–51. doi: 10.1177/00037028211056975 (PMC8915227; doi:10.1177/00037028211056975)
Supplement: sj-pdf-1-asp-10.1177_00037028211056975 – Supplemental Material for Investigating the Performances of Wide-Field Raman Microscopy with Stochastic Optical Reconstruction Post-Processing [file sj-pdf-1-asp-10.1177_00037028211056975.pdf]

Supplementary Material: Investigating the performances of wide-field Raman microscopy with stochastic optical reconstruction post-processing

*Leila Mazaheri, Joachim Jelken, Maria O. Avilés, Sydney Legge, François Lagugné-Labarthet**

The University of Western Ontario (Western University), Department of Chemistry, 1151 Richmond St., London, Ontario, Canada, N6A 5B7

Corresponding Author

François Lagugné-Labarthet, Department of Chemistry, Western University, 1151 Richmond Street, London, ON, N6A 5B7, Canada. Email: flagugne@uwo.ca

Raman-STORM of silicon structures:

Optimal reconstruction parameters for higher localization and photon count precision depend on the number of photons per pixel and the pixel size.[1-3] The main key fitting parameters in the Rapid-STORM analysis for evaluating photons per pixel are: i) the minimum spot distance (minimum distance between the maximum of two gaussian PSFs, with a constant FWHM defined by the optical system and set to 500 nm in the software, which corresponds to ~4 pixel of the EMCCD) and ii) the intensity threshold (minimum intensity until which fluctuations are being considered). In Figure 3a-i, these parameters are tuned to obtain the highest contrast in the Raman image. The intensity scale bar in Raman-STORM is expressed in photon counts. For different minimum distances, different set of intensity thresholds were studied. For higher value of the minimum spot distance, higher intensity threshold is required to get similar contrast and details of the reconstructed image. For higher value of intensity threshold, more spots with weaker signals are dismissed, and only spots with higher intensity fitted with the centroid localization algorithms are shown. Noise due to background become dominant for fewer photon counts per pixel. The intensity threshold and minimum spot distance for initiation of localization should be tuned depending on experimental conditions. For the experimental condition in this work, for smaller minimum spot distance, more photon counts, and higher contrast are obtained. For example, a comparison between Figures S1a, d, and g, highlight that Figure S1a has the highest contrast. On this sample the interest was to localize higher intensity Si signals and dismiss the lower intensity, hence minimum spot distance of 1 pixel (~ 130 nm, dependent on the telescope used in the setup) and threshold intensity of 5 yield a more contrasted image.

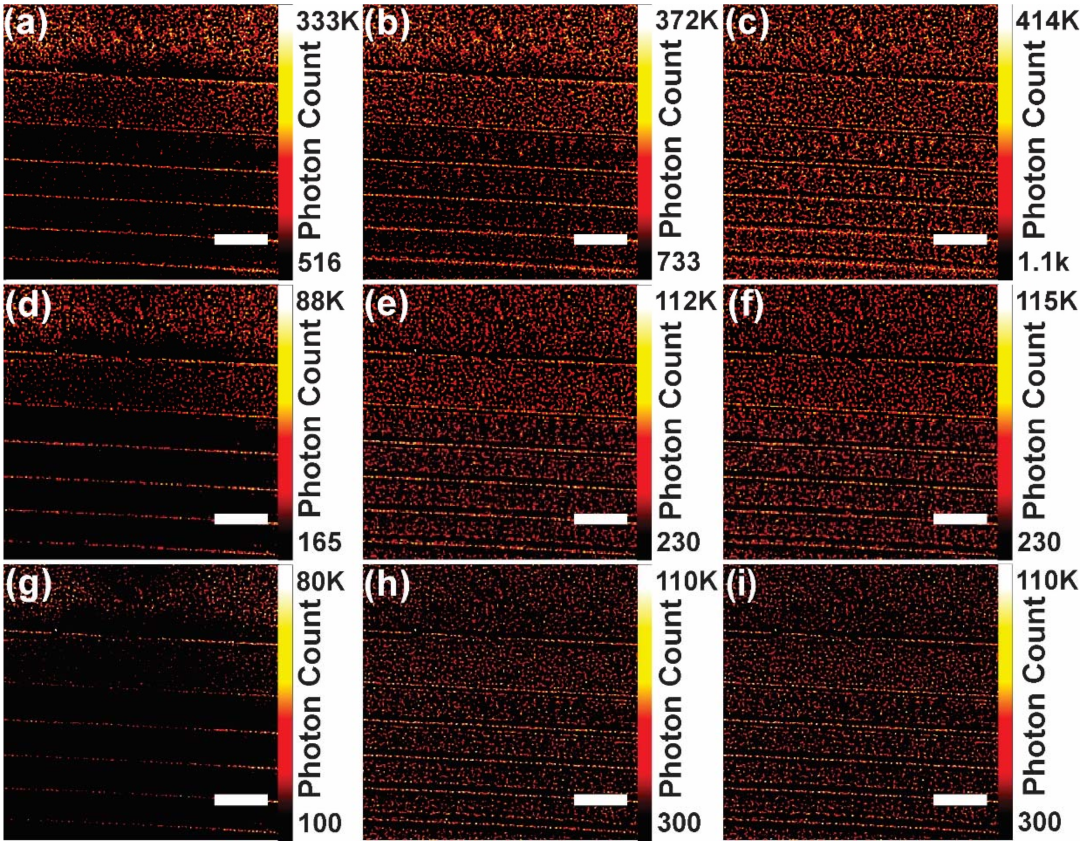

Figure S1. A series of five-hundred images were captured and treated with Rapid-STORM to build a higher resolution image. The scale bar on images represents 20 μm. For a fixed minimum spot distance, different intensity threshold has been tried: a-c) minimum spot distance of 1 pixel, intensity threshold of 5, 3, 1, d-f) minimum spot distance of 3 pixel, intensity threshold of 50, 15, 3, g-j) minimum spot distance of 5 pixel, intensity threshold of 100, 50, 20 respectively.

Raman-STORM of polystyrene microspheres.

In Figure S2, the corresponding spectrum for wide and narrow setting is presented. For the LCTF set in wide setting, the spectral domain is set to a width of 30 nm corresponding to a spectral range of [525-1466] cm^{-1} highlighted in Figure S2a. Corresponding STORM images presented in Figure S2c reconstructed from a series of 100 images with a total acquisition time of 28 s. On the other hand, in narrow setting (10 nm ([844-1160] cm^{-1})) mainly the second phonon mode of Si in range

of 930 and 1030 cm^{-1} and the peak at 1000 cm^{-1} of PS associated with ring breathing and in plane CH deformation were captured (Fig. S2a and S2d). Corresponding STORM image reconstructed from a series of 100 images is illustrated in Fig. S2e. In the both cases of wide and narrow settings, the reconstructed images have higher contrast and enhanced spatial resolution compared to the optical images.

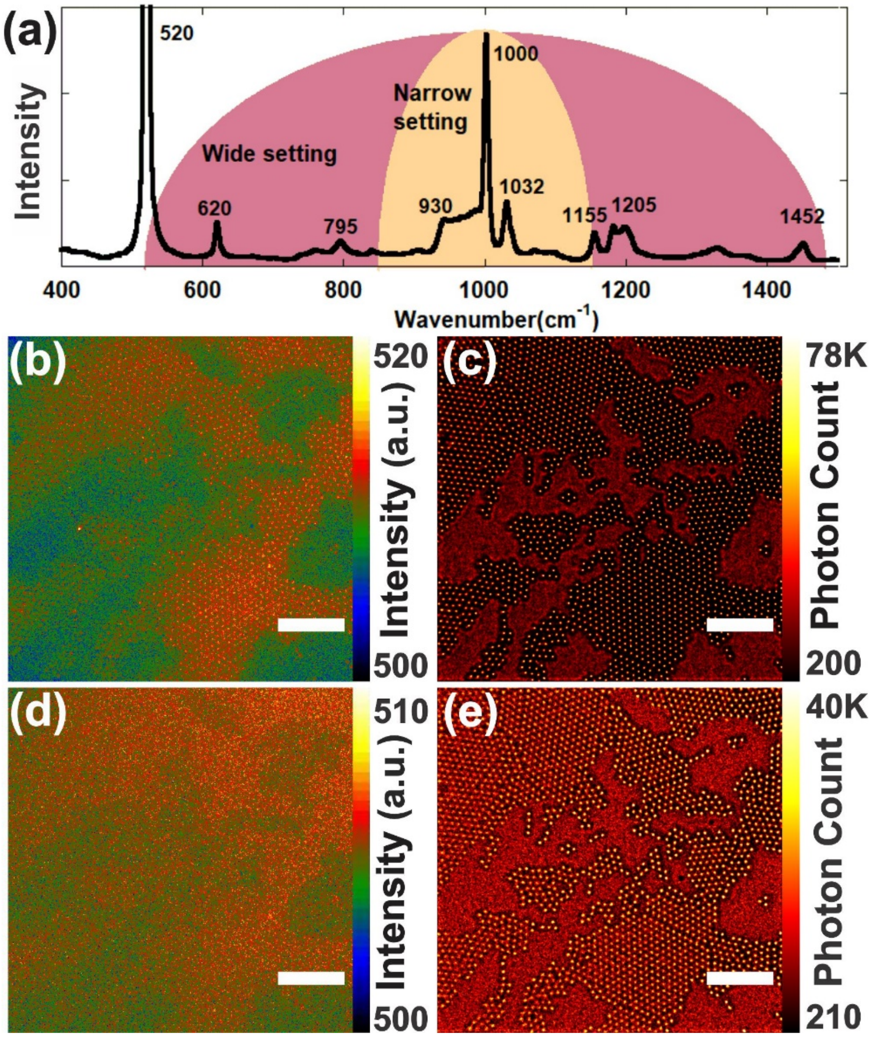

Figure S2. a) Spectrum of the PS microspheres in the [400-1500] cm^{-1} range. The spectral range corresponding to the wide and narrow settings of the LCTF are indicated for central wavelength of 562 nm (1004 cm^{-1}) b) Wide-field Raman imaging of two micrometer PS microspheres on Si substrate for wide setting ([525-1466] cm^{-1}) and its corresponding c) Rapid-STORM image

reconstructed from a series of 100 images. d) Wide field Raman image of the PS microspheres for narrow setting ([844-1160] cm^{-1}) and its corresponding e) STORM image reconstructed from a series of 100 images. The scale bar represents 20 μm.

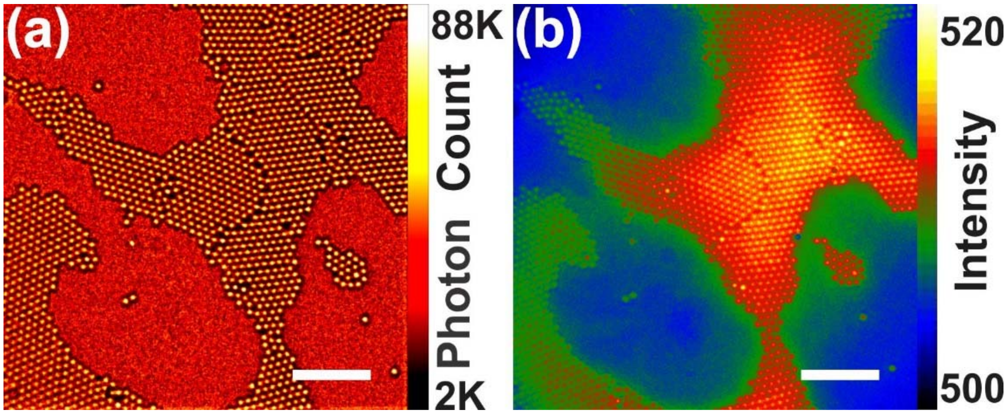

Figure S3. a) Rapid-STORM treated image of a kinetic series of five hundred images vs b) average image of accumulation of five hundred images (140 s acquisition time).

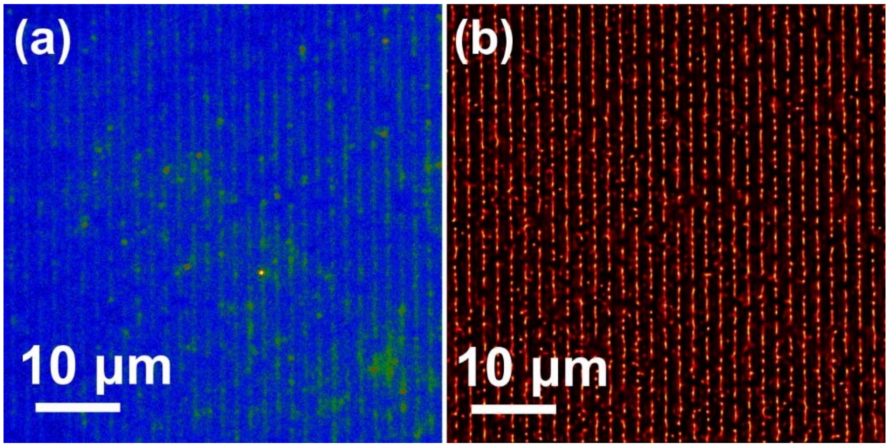

Figure S4. a) single wide-field image of Au nanoraspberries organized along parallel lines separated by 1.67 µm. Image was acquired in 0.28 s. b) STORM image of the same sample and location obtained from a series of 500 images acquired in 280 s.

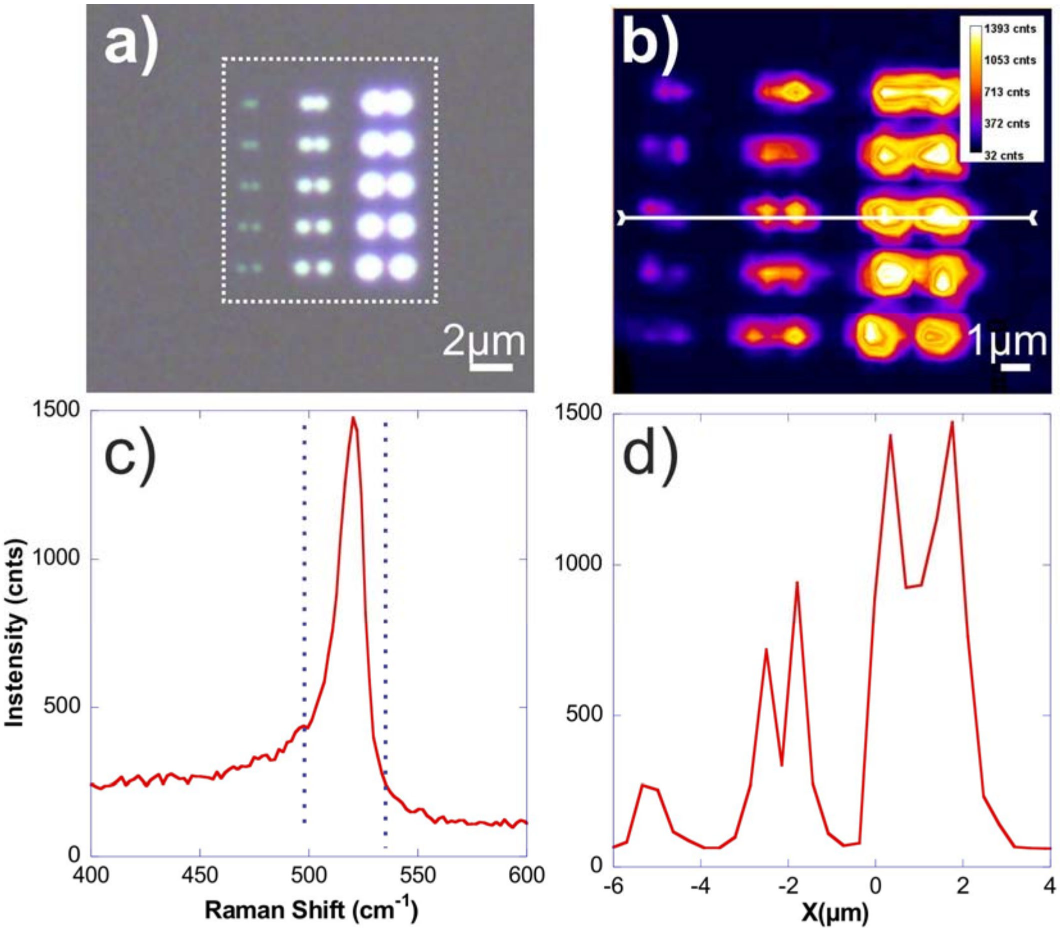

Figure S5. a) Video image of the reference sample. From bottom to top, Right to left: Si disks over glass with diameters of 1µm, 500 and 200 nm separated by 500, 400, 300, 200 and 100 nm edge-to-edge gap distance. b) Raman map integrated over the silicon band [494-532] cm^{-1}. The map is comprised of 30×30 spectra and a spacing of 0.4 µm along the x and y directions was used. Grating of 1200 gr/mm, pinhole of 300 µm and slit of 100 µm were used. Total acquisition time of 12 minutes. c) Typical spectrum collected in 0.5 s acquisition time. d) cross section along the structures separated by a 300 nm gap.

References

1. S. Van de Linde, A. Löschberger, T. Klein, et al. "Direct Stochastic Optical Reconstruction Microscopy with Standard Fluorescent Probes". Nat. Protoc. 2011. 6(7): 991-1009.
2. S. Wolter. An Accurate and Efficient Algorithm for Real-Time Localisation of Photoswitchable Fluorophores. [Diploma Thesis], Bielefeld, Germany: Bielefeld University, 2009.
3. R. E. Thompson, D. R. Larson, W. W. Webb. "Precise Nanometer Localization Analysis for Individual Fluorescent Probes". Biophys. J. 2002. 82(5): 2775-2783.
